# Study on the improvement of electroencephalogram signal characteristics in stroke patients by edaravone dexborneol

**DOI:** 10.3389/fnins.2026.1772921

**Published:** 2026-03-17

**Authors:** Song Zhang, Honglei Jiao

**Affiliations:** Department of Neurology, The Second Hospital of Hebei Medical University, Shijiazhuang, China

**Keywords:** cerebral infarction, complexity, edaravone dexborneol, electroencephalogram (EEG), entropy, neurological recovery, power spectrum

## Abstract

**Objective:**

To investigate the effects of edaravone dexborneol injection on the cortical electroencephalogram (EEG) signal characteristics in patients with acute ischemic stroke (cerebral infarction) and evaluate its mechanism in promoting neurological recovery from a neurophysiological perspective.

**Methods:**

A total of 80 patients with acute anterior circulation cerebral infarction, treated at the Department of Neurology, Second Hospital of Hebei Medical University, were randomly divided into the control group (routine treatment) and the experimental group (routine treatment + edaravone dexborneol). EEG data were collected at rest for both groups at baseline (T0) and 7 days after treatment (T1). The collected EEG signals were pre-processed, and the relative power spectral densities in the delta, theta, alpha, and beta frequency bands of the affected hemisphere were calculated and compared between the two groups. Additionally, signal complexity (Lempel–Ziv complexity, LZC) and approximate entropy (ApEn) were assessed. Neurological deficits were evaluated using the National Institutes of Health Stroke Scale (NIHSS), and the correlation between EEG characteristics and NIHSS scores was analyzed.

**Results:**

After treatment, compared to the control group, the experimental group showed a significant decrease in the relative power in the delta frequency band and a significant increase in the relative power in the alpha and beta frequency bands in the affected hemisphere (*p* < 0.05). The LZC and ApEn values of the EEG signals in the experimental group were significantly higher than those in both the baseline and the control group post-treatment (*p* < 0.05). The NIHSS score improvement in the experimental group was significantly greater than that in the control group (*p* < 0.05). Furthermore, the alpha/beta power ratio, LZC, and ApEn values were significantly positively correlated with the improvement in NIHSS scores.

**Conclusion:**

Treatment with edaravone dexborneol can effectively improve abnormal EEG patterns in patients with cerebral infarction, characterized by reduced slow-wave activity and increased fast-wave activity and EEG complexity/orderliness. These beneficial changes in EEG characteristics are synchronized with clinical neurological recovery, suggesting that the drug may promote the reshaping and functional reorganization of neural networks by reducing oxidative stress, neuroinflammation, improving cerebral microcirculation, and enhancing neuronal metabolism.

## 1 Introduction

Acute ischemic stroke (cerebral infarction) is the leading cause of death and disability in adults (GBD 2019 Stroke Collaborators, 2021). Its pathological core is the interruption of local cerebral blood flow, leading to neuronal ischemia and hypoxic damage, which in turn triggers a cascade of oxidative stress, inflammatory response, and cell apoptosis, ultimately causing irreversible neurological deficits (Khoshnam et al., 2017; Tuo et al., 2022). Neurological rehabilitation depends not only on structural protection but also on the functional plasticity of neural networks.

Electroencephalography (EEG), as a non-invasive, real-time, and high temporal resolution neurophysiological monitoring technique, can directly reflect the synchronized electrical activity of cortical neurons (Delcamp et al., 2024). After a cerebral infarction, neuronal dysfunction or network disconnection in the ischemic core and surrounding penumbra area manifests as characteristic changes in EEG: an increase in slow-wave activity (delta and theta waves), and a decrease in fast-wave activity (alpha and beta waves) that reflect normal cortical function and cognitive activity (Wu et al., 2016; Ferreira et al., 2021). Additionally, the complexity (such as Lempel–Ziv complexity) and entropy (such as approximate entropy) of EEG signals are reduced, reflecting decreased efficiency in neural information processing and the loss of system orderliness (Sun et al., 2017; Sun et al., 2021). Therefore, EEG characteristics can serve as sensitive indicators for assessing the severity of brain injury and monitoring rehabilitation progress.

Edaravone is a potent free radical scavenger, while dexborneol has dual effects of anti-inflammatory action and improving microcirculation (Watanabe et al., 2004; Wang et al., 2023). The combination of both in a compound formulation (edaravone dexborneol) theoretically exerts a synergistic neuroprotective effect, which has been shown in clinical studies to outperform single-agent edaravone (Xu et al., 2021; Huang et al., 2022). However, evaluations of its efficacy are primarily based on clinical scales (such as NIHSS, mRS), lacking objective and quantitative neurophysiological evidence. Although these scales are the gold standard in stroke research, they possess a degree of subjectivity and struggle to directly reflect the drug’s impact on cerebral cortical neuroelectrical activity and network function. To date, there is a lack of studies utilizing EEG, an objective neurophysiological indicator, to quantitatively assess the effect of edaravone dexborneol on brain function in patients with acute ischemic stroke. This study aims to quantitatively reveal the improvement effect of edaravone dexborneol on the damaged brain neural network function by analyzing high-resolution EEG signals before and after treatment in patients with cerebral infarction, from multiple dimensions such as power spectrum, complexity, and entropy values. Compared to traditional clinical scale assessments, EEG, as a non-invasive, high-temporal-resolution neurophysiological technique, can directly and objectively quantify changes in cerebral cortical function. Introducing EEG into the efficacy evaluation of edaravone dexborneol not only provides direct evidence of the drug’s effect on neural functional networks but also holds the potential to reveal the neurophysiological mechanisms underlying drug-promoted neurological recovery. This represents an important complement and deepening of existing research primarily based on clinical scales and will provide a deeper electro-physiological basis for its clinical application. Therefore, this study aims to investigate the effect of edaravone dexborneol on cortical EEG signal characteristics in patients with acute anterior circulation cerebral infarction. We hypothesize that, compared to conventional treatment alone, the addition of edaravone dexborneol will more significantly improve abnormal EEG patterns (manifested as reduced slow-wave activity, increased fast-wave activity, and enhanced EEG complexity), and that these neurophysiological improvements will correlate with the reduction in clinical neurological deficit severity (decrease in NIHSS score).

## 2 Methods

### 2.1 Study subjects

Patients with acute anterior circulation cerebral infarction who were admitted to the Department of Neurology at our hospital from January 2021 to December 2024 were selected for the study.

*Inclusion criteria*: First onset of stroke, diagnosed as acute infarction in the unilateral middle cerebral artery territory based on head MRI (DWI sequence). Onset time within 48 h. NIHSS score between 5 and 20 points. Age between 40 and 80 years. Signed informed consent from the patient or their family members.

*Exclusion criteria*: Hemorrhagic stroke or other intracranial lesions. History of severe neurological disorders or epilepsy. Severe heart, liver, or kidney dysfunction. Allergy to the study drug. Inability to cooperate with EEG examination.

A total of 80 patients were finally enrolled with 40 patients in each group. They were randomized using a computer-generated random number sequence. This sequence was generated and sealed in opaque envelopes by a researcher not involved in patient recruitment or assessment. After obtaining informed consent, a third-party nurse opened the envelopes to assign the group allocation. There were no statistically significant differences between the two groups in terms of age, gender, infarct side, or baseline NIHSS scores (*p* > 0.05), indicating comparability.

Ethical approval for this study was obtained from the Ethics Committee of the Second Hospital of Hebei Medical University. Written informed consent was obtained from all participants prior to enrollment.

### 2.2 Treatment methods

*Control group*: Received routine treatment recommended by the “Guidelines for the Diagnosis and Treatment of Acute Ischemic Stroke (Version for China),” which included antiplatelet aggregation (aspirin or clopidogrel), statins for lipid regulation and plaque stabilization, blood pressure and blood glucose control, and rehabilitation training.

*Experimental group*: On the basis of routine treatment, received edaravone dexborneol injection (specification: edaravone 30 mg + dexborneol 7.5 mg/vial). Administration: 1 vial per dose, twice daily via intravenous infusion, with each infusion lasting no less than 30 min. The treatment was given continuously for 7 days.

### 2.3 EEG signal collection and preprocessing

*Collection equipment*: A 32-channel EEG system (nicolet) was used, with a sampling frequency set to 1,000 Hz and a bandwidth of 0.5–70 Hz.

*Collection procedure*: The recordings were performed in a quiet, dimly lit shielded room. The patient was positioned supine, with eyes closed and relaxed, while staying awake. Resting-state EEG signals were collected at two time points: within 24 h before the treatment began (T0) and within 24 h after the treatment ended (T1). Each recording session lasted for 20 min.

*Preprocessing*: Offline analysis was performed using MATLAB R202Xa and the EEGLAB toolbox.

The steps included: Manually removing segments with obvious muscle activity or eye movement artifacts. Applying a bandpass filter between 1–45 Hz. Re-referencing to the average reference. Using independent component analysis (ICA) to remove residual eye and cardiac artifacts. After preprocessing, five segments of artifact-free and stable data, each lasting 4 s (totaling 20 s), were selected for further analysis. The analysis focused on the central area (C3/C4), parietal area (P3/P4), and occipital area (O1/O2) of the affected hemisphere.

All preprocessing of EEG data (artifact removal, filtering, ICA decomposition) and subsequent calculation of feature values (power spectrum, LZC, ApEn) were performed by a researcher who was blinded to the clinical group allocation.

### 2.4 EEG signal feature analysis

This study focused on analyzing EEG signals from the central (C3/C4), parietal (P3/P4), and occipital (O1/O2) regions of the affected hemisphere. The selection rationale is as follows: The central region covers the sensorimotor cortex, directly relevant to the common sensorimotor deficits in the included stroke patients. The parietal cortex is involved in spatial attention and sensory integration, making it a key region for post-stroke cognitive impairment. The occipital region, as the visual cortex, is sensitive to ischemia and hypoxia, and its stable alpha rhythm is a classic indicator for assessing brain functional status. Analyzing these regions allows for a multi-dimensional evaluation of the drug’s effect on cortical function, encompassing sensorimotor, cognitive, and fundamental rhythm aspects.

#### 2.4.1 Power spectral density analysis

The Welch method (window length 1 s, 50% overlap) was used to calculate the power spectrum of the EEG signals from each electrode. The spectrum was divided into four classic frequency bands: delta (1–4 Hz), theta (4–8 Hz), alpha (8–13 Hz), beta (13–30 Hz). The relative power spectral density (rPSD) of each frequency band was calculated, which is the ratio of the power in that frequency band to the total power (1–30 Hz).

#### 2.4.2 Complexity analysis (Lempel–Ziv complexity, LZC)

LZC is an effective method for measuring the randomness or complexity of a time series. A higher value indicates a more complex signal pattern and richer information content. The EEG time series after preprocessing was binarized, and the LZC value was then calculated.

#### 2.4.3 Entropy analysis (approximate entropy, ApEn)

ApEn is used to measure the regularity and unpredictability of a time series. In a physiological context, highly regular, repetitive patterns (such as periodic slow waves under severe pathological conditions) correspond to lower ApEn values, indicating a state of low complexity and high predictability in the system. Conversely, healthy, active EEG signals contain more aperiodic, complex fluctuations, and their ApEn values are higher, signifying richer and more unpredictable system dynamics (not mere “randomness,” but structured complexity). Therefore, an increase in ApEn is typically interpreted as an enhancement in the brain’s capacity for information processing or dynamic flexibility. The embedding dimension was set to *m* = 2, and the tolerance was set to *r* = 0.2 times the standard deviation of the sequence to calculate the ApEn value.

### 2.5 Clinical efficacy assessment

The NIHSS scores were evaluated by a neurologist who was blinded to the group assignments at both T0 and T1. The change in NIHSS scores (ΔNIHSS = NIHSS_T0 − NIHSS_T1) was calculated to assess clinical improvement. NIHSS scores were assessed at time points T0 and T1 by a neurologist who was blinded to the treatment group allocation.

### 2.6 Statistical analysis

Statistical analysis was performed using SPSS 26.0 software. Continuous data were presented as mean ± standard deviation. Intra-group comparisons before and after treatment were conducted using paired *t*-tests; inter-group comparisons were performed using independent samples *t*-tests. Categorical data were analyzed using the chi-square test. The correlation between EEG features and ΔNIHSS was analyzed using Pearson correlation analysis. A *p*-value of <0.05 was considered statistically significant. For comparisons involving multiple sets of EEG features (different frequency bands, different electrodes, and different complexity measures), the false discovery rate (FDR) method was used for multiple comparison correction to control the false positive rate. The FDR-corrected *p*-values (*q*-values) are reported.

## 3 Results

### 3.1 Power spectral density changes

All patients completed the 7-day treatment and evaluation, with no loss to follow-up or withdrawals. During the treatment period, 2 patients in the experimental group developed mild liver function abnormalities, and 1 patient in the control group reported gastrointestinal discomfort. All events resolved after symptomatic treatment, and no serious adverse events occurred. There was no statistically significant difference in the incidence of adverse events between the two groups.

**Figure 1:**
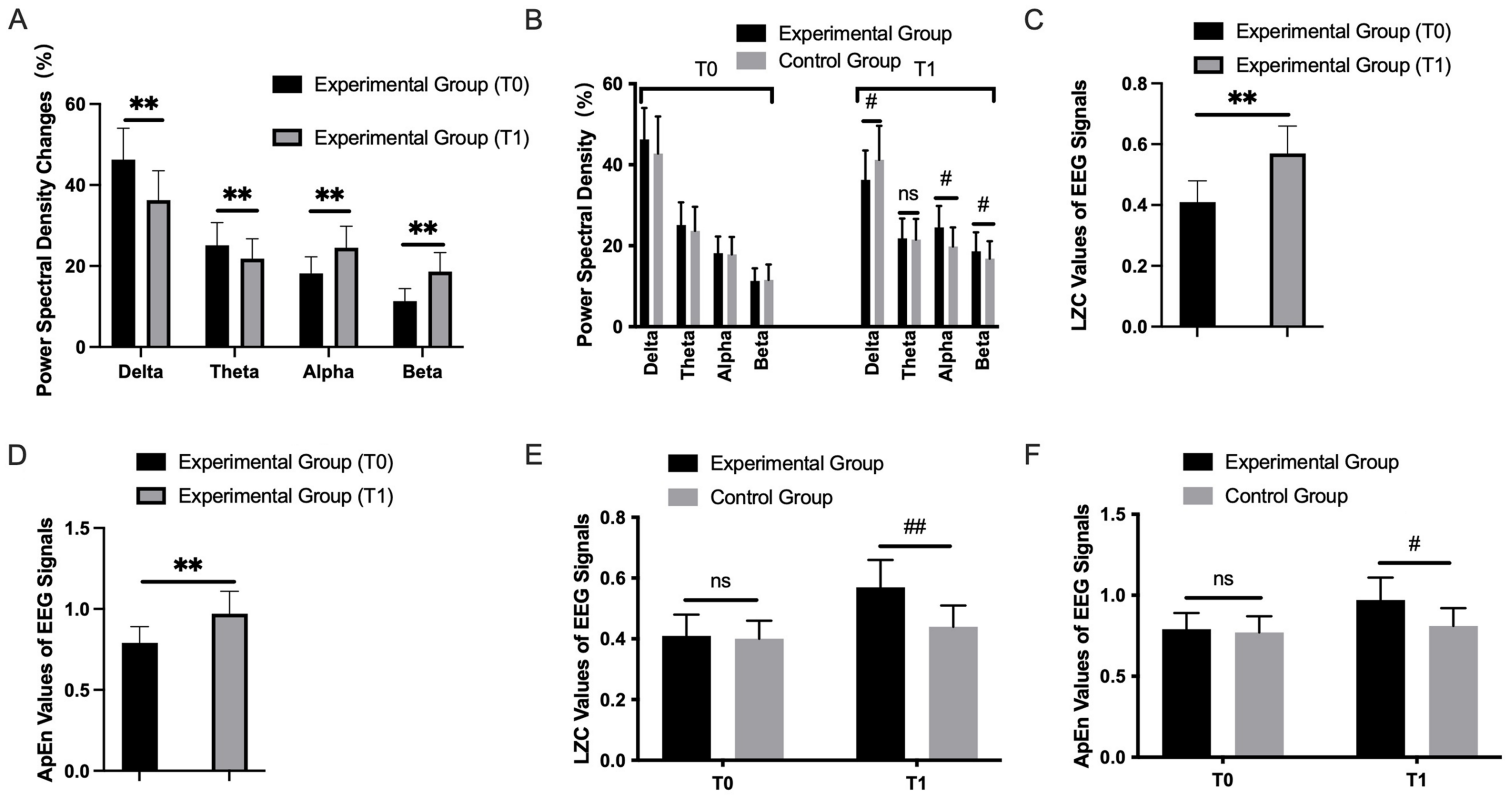
**(A)** Comparison of rPSD of each frequency band in the affected central region (C3/C4) at T1 in the experimental group. **(B)** Comparison of rPSD of each frequency band before and after treatment between the two groups. ^#^*p* < 0.05 and ^##^*p* < 0.01. Comparison of LZC **(C)** and ApEn **(D)** values of EEG signals in experimental group at the two time points, **(E,F)** between the two groups of patients. ^*^p < 0.05 and ^**^p < 0.01; ^#^*p* < 0.05 and ^##^*p* < 0.01.

Power spectral density analysis revealed significant improvements in the experimental group after treatment (Table 1 and Figure 1A). Compared to baseline, the experimental group showed significantly reduced relative power in the delta (*p* < 0.01) and theta (*p* < 0.01) bands, alongside significantly increased relative power in the alpha (*p* < 0.01) and beta (*p* < 0.01) bands in the affected hemisphere. In contrast, the control group exhibited only a slight, non-significant decrease in theta power (*p* > 0.05), with no significant changes in other frequency bands.

**Table 1:** Comparison of relative power spectrum density of each frequency band in the affected central region (C3/C4) before and after treatment in two groups of patients (mean ± standard deviation, %)^*^.

Group	Time	Delta	Theta	Alpha	Beta
Experimental group (*n* = 40)	T0	46.3 ± 7.7	25.1 ± 5.6	18.2 ± 4.1	11.3 ± 3.1
	T1	36.3 ± 7.2^**#^	21.8 ± 4.9^**^	24.5 ± 5.3^**#^	18.6 ± 4.7^**#^
Control group (*n* = 40)	T0	42.8 ± 9.1	23.7 ± 5.9	17.9 ± 4.3	11.6 ± 3.8
	T1	41.2 ± 8.4	21.5 ± 5.1	19.8 ± 4.7	16.8 ± 4.3

Compared with T0 in the same group, ^*^p < 0.05 and ^**^p < 0.01; compared with T1 in the control group, ^#^*p* < 0.05 and ^##^*p* < 0.01.

Between-group comparisons at T1 further confirmed these findings (Figure 1B). The experimental group demonstrated significantly lower delta power (*p* < 0.05) and significantly higher alpha and beta power (*p* < 0.05 for both) in the affected hemisphere compared to the control group. No significant between-group difference was observed in the theta band (*p* > 0.05). Notably, no significant power changes were detected in the healthy hemisphere in either group, suggesting a localized treatment effect on the affected cortex.

### 3.2 Changes in complexity and entropy values

Next, we analyzed the changes in complexity and entropy values of the EEG signals. As shown in Table 2, after treatment, the Lempel–Ziv complexity (LZC) value of the affected hemisphere in the experimental group significantly increased (*p* < 0.01) (Figure 1C). An increase in LZC typically indicates greater complexity in the EEG signal, reflecting more organized and flexible brain activity. This suggests that the recovery of brain function in the experimental group after treatment is not only reflected in changes in power spectral density but also accompanied by an increase in signal complexity. After treatment, the approximate entropy (ApEn) value of the affected hemisphere in the experimental group significantly increased (*p* < 0.01) (Figure 1D). The increase in ApEn value represents a reduction in the randomness and unpredictability of the EEG activity, meaning the coordination of the neural network has improved, which is closely associated with the recovery of neural function. In the control group, there were no significant changes in the LZC and ApEn values before and after treatment (*p* > 0.05) (Figures 1E,F), indicating that the complexity and entropy of the EEG signals did not change significantly, suggesting that the treatment effect was relatively poor in the control group.

**Table 2:** Comparison of LZC and ApEn values of EEG signals in the affected brain region (mean ± standard deviation) between the two groups of patients.

Group	Time	LZC	ApEn
Experimental group (*n* = 40)	T0	0.41 ± 0.07	0.79 ± 0.10
	T1	0.57 ± 0.09^**##^	0.97 ± 0.14^**#^
Control group (*n* = 40)	T0	0.40 ± 0.06	0.77 ± 0.10
	T1	0.44 ± 0.07	0.81 ± 0.11

Compared with T0 in the same group, ^*^p < 0.05 and ^**^p < 0.01; compared with T1 in the control group, ^#^*p* < 0.05 and ^##^*p* < 0.01.

### 3.3 Changes in clinical neurological function scores

We evaluated the changes in clinical neurological function scores. As shown in Table 3, after treatment, the NIHSS scores of both groups significantly decreased compared to pre-treatment, indicating improvement in the neurological function of both groups. The ΔNIHSS score change in the experimental group was (7.2 ± 2.1) which was significantly higher than the ΔNIHSS score in the control group (4.5 ± 1.8) (*p* < 0.01). This result suggests that the improvement in neurological function in the experimental group was significantly better than in the control group, supporting the superiority of the treatment effect in the experimental group.

**Table 3:** The NIHSS scores of the two patient groups were compared at baseline and on day 7 of treatment, with results presented as mean ± standard deviation.

Group	NIHSS (T0)	NIHSS (T1)	ΔNIHSS
Experimental group (*n* = 40)	13.0 ± 3.2	6.0 ± 3.1	7.2 ± 2.1^**^
Control group (*n* = 40)	12.5 ± 2.5	8.0 ± 3.0	4.5 ± 1.8^**^

Compared with T0 in the same group, ^**^p < 0.01.

### 3.4 Correlation analysis

Finally, we conducted a Pearson correlation analysis between EEG signals and behavioral outcomes. We found that, in the experimental group, the alpha/beta power ratio, LZC value, and ApEn value of the affected hemisphere at T1 were all significantly positively correlated with ΔNIHSS scores (*r* values of 0.632, 0.581, and 0.524, respectively, all *p* < 0.01). This means that the stronger the fast-wave (alpha/beta wave) power, the higher the signal complexity (LZC value) and entropy (ApEn value), the more significant the improvement in the patient’s neurological function. These results indicate that enhanced neural activity, more complex signals, and higher entropy values are closely associated with the clinical improvement in patients.

## 4 Discussion

In the initial days following an acute ischemic stroke, oxidative stress and inflammatory responses in the peri-infarct tissue are most active, serving as the primary causes of aggravated brain tissue damage. Therefore, early neuroprotective intervention represents a critical time window for mitigating neurological deficits. Edaravone dexborneol is a neuroprotective agent that primarily targets oxidative stress and inflammatory responses during ischemia–reperfusion injury (Xu et al., 2021). Based on the treatment course recommended in the drug’s prescribing information approved in China for acute ischemic stroke, as well as the conventional treatment duration for cerebral infarction currently adopted in China, a 7-day course has been proven to be effective and safe. This study, through quantitative electroencephalogram (EEG) analysis, provides the first multidimensional electrophysiological evidence confirming the beneficial effects of edaravone dexborneol on impaired brain function in patients with cerebral infarction. The significant differences in EEG characteristics observed between the experimental and control groups are closely related to the multi-target neuroprotective mechanisms of the drug. Based on the known pharmacological effects of edaravone dexborneol, we speculate that the aforementioned EEG improvements may be associated with the following mechanisms:

### 4.1 Regulation of EEG rhythms

The results showed that, after treatment, the experimental group exhibited significant suppression of pathological slow-wave (delta wave) activity in the affected hemisphere, while physiological fast-wave (alpha, beta waves) activity was significantly enhanced. An increase in delta wave activity is typically associated with cortical neuron dysfunction, reduced metabolism, and blood–brain barrier disruption (Ferreira et al., 2021). Alpha/Beta waves are fundamental rhythms that maintain normal cognitive, sensory-motor, and attention functions (Cannon et al., 2014). The “normalization” of EEG rhythms after edaravone dexborneol combined treatment may reflect the protective effect of the drug on neuronal function in the ischemic penumbra. Edaravone scavenges hydroxyl radicals and other toxic oxidative byproducts, reducing lipid peroxidation damage in neurons (Ren et al., 2015). Dexborneol, by inhibiting inflammatory pathways such as nuclear factor-kappa B (NF-κB), mitigates the cascade of neuroinflammation (Liu et al., 2011). The synergistic effects of both drugs stabilize the neuronal membrane potential, improve mitochondrial energy metabolism, and thus promote the recovery of synchronized oscillatory activity in cortical neurons (Yang et al., 2022; Zhang et al., 2025).

### 4.2 Effect on EEG complexity and information entropy

LZC and ApEn are nonlinear indicators used to assess the dynamics of EEG. After cerebral infarction, a large number of neurons die or undergo functional decompensation, leading to sparse neural network connections and decreased information transfer efficiency (Liu et al., 2016; Tuo et al., 2022). This manifests as EEG signals becoming simpler and more regular (with lower complexity and entropy values). This study found that patients in the experimental group exhibited significantly increased LZC and ApEn values after treatment. The increase in LZC directly indicates heightened complexity in EEG signal patterns. The increase in ApEn, based on its definition, suggests reduced regularity and increased unpredictability of the EEG sequence. Together, these findings point to an expansion of the neural network’s dynamic range and an enhancement of its information processing capacity (Sun et al., 2017; Cheng et al., 2019). This change may be related to the neural network, under the protection of the medication, shifting from a pathological, highly synchronized low-energy state (low entropy) to a more flexible and adaptable functional state (high entropy) (Chen et al., 2022; Hu et al., 2022).

### 4.3 Correlation between EEG features and clinical rehabilitation

In this study, the improvements in EEG features (alpha/beta power, LZC, ApEn) were significantly positively correlated with improvements in NIHSS scores. This strongly demonstrates that objective changes in EEG indicators can effectively reflect the extent of recovery in subjective clinical neurological function. This provides a basis for future use of quantitative EEG as an objective biomarker for assessing stroke rehabilitation efficacy.

Potential reasons for the significant differences between the experimental and control groups include the fact that edaravone dexborneol is a potent free radical scavenger. Following cerebral ischemia, a burst of free radicals leads to oxidative damage in neurons, vascular endothelial cells, and glial cells, exacerbating cerebral edema and inflammatory responses. By scavenging hydroxyl radicals and inhibiting lipid peroxidation, edaravone dexborneol alleviates oxidative stress damage, creating a more favorable microenvironment for neuronal survival (Huang et al., 2022). This may serve as the pathophysiological basis for the reduction in pathological slow waves (such as *δ* and *θ* waves) observed on EEG. After entering brain tissue, edaravone dexborneol may improve microcirculation and reduce cerebral edema, thereby aiding in the rescue of the ischemic penumbra and promoting the recovery of neural electrical activity. Edaravone dexborneol may also protect neuronal and synaptic function, reduce excitotoxicity, and stabilize cell membranes (Xu et al., 2021). The increase in EEG complexity and order reflects the improvement in the overall function and information processing capacity of neural networks, which may be a direct manifestation of the protection of neural units and their connections.

### 4.4 Potential influencing factors and generalizability of the study findings

The study population was limited to patients with acute anterior circulation ischemic stroke and moderate neurological deficits. Therefore, the conclusions are primarily applicable to this specific group. Factors such as infarct location (e.g., cortical vs. subcortical), lesion volume, collateral circulation compensation, and the intensity of individualized rehabilitation therapy received by the patients may all influence the patterns of EEG activity recovery and the response to the medication. For instance, patients with large-area infarcts or brainstem infarcts might exhibit different electrophysiological change trajectories. Future studies are needed to validate the generalizability of these findings in broader stroke subtypes (e.g., posterior circulation infarction, mild or severe stroke) and to explore clinical and imaging predictors influencing EEG reactivity.

### 4.5 Limitations and future perspectives

First, although blinding was implemented for clinical assessors and EEG data analysts, due to the nature of the treatment medication, it was not feasible to blind the patients and the medical staff administering the treatment (non-double-blind design), which may introduce performance bias. We attempted to mitigate its impact on the results by using objective EEG quantitative measures and blinded assessments.

Second, to maximize the exclusion of artifact interference, this study ultimately selected only 20 s of high-quality resting-state EEG data for analysis. While this ensures a reliable signal basis for feature calculation, the relatively short duration may not fully capture all nonlinear dynamic properties of the brain’s resting-state networks. Future research could consider extending the analysis duration or employing dynamic analysis techniques to more comprehensively assess the time-varying characteristics of EEG activity.

Third, this study primarily observed phenomena from an electrophysiological perspective. Although reasonable speculation about potential mechanisms was made based on pharmacology, it did not synchronously measure serum biomarkers such as oxidative stress and inflammatory factors, or imaging changes like functional magnetic resonance imaging. Therefore, the direct causal relationship between the improvement in EEG features and specific molecular or vascular mechanisms still requires confirmation through future multimodal studies.

Fourth, this study only evaluated the EEG and clinical effects immediately after the treatment course (14 days). It remains unclear whether these early-improved electrophysiological patterns can be maintained in the long term and ultimately translate into better long-term functional outcomes (such as the 3-month modified Rankin scale score). Future studies with longer-term follow-up are needed to clarify the predictive value of early neuroelectrophysiological remodeling induced by edaravone dexborneol for long-term prognosis.

In summury, this study provides the first quantitative EEG evidence for the therapeutic effect of edaravone dexborneol in acute ischemic stroke. Our results confirm that the addition of edaravone dexborneol to conventional treatment significantly ameliorates abnormal EEG patterns in patients, characterized by reduced slow-wave activity, increased fast-wave activity, and enhanced signal complexity. Moreover, these objective neurophysiological improvements occurred in parallel with the recovery of clinical neurological function. This finding fills a critical gap in the efficacy evaluation of this drug, which has previously lacked quantitative neurophysiological evidence, and offers a novel perspective for understanding the mechanisms underlying its promotion of neurological recovery.

## 5 Conclusion

This study confirms that the addition of edaravone dexborneol to standard treatment significantly improves the abnormal EEG patterns in patients with acute cerebral infarction. Specifically, it involves the suppression of pathological slow-wave activity, enhancement of physiological fast-wave activity, and an increase in EEG signal complexity and information entropy. These beneficial changes in EEG features are closely associated with improvements in the clinical neurological deficits of the patients. From a neurophysiological perspective, it objectively reveals that edaravone dexborneol promotes the reorganization and repair of impaired neural network function through its multi-target neuroprotective mechanisms. Quantitative EEG analysis can serve as an effective auxiliary tool for evaluating the therapeutic efficacy of the drug and monitoring the progress of neurological rehabilitation.

## Data Availability

The datasets generated and/or analyzed during the current study are not publicly available due to patient privacy and confidentiality concerns, as mandated by the research ethics committee. However, they are available from the corresponding author on reasonable request, subject to a formal data sharing agreement. Requests to access these datasets should be directed to Honglei Jiao, eglantine_med@hotmail.com.

## References

[ref1] Cannon J. McCarthy M. M. Lee S. Lee J. Börgers C. Whittington M. A. . (2014). Neurosystems: brain rhythms and cognitive processing. Eur. J. Neurosci. 39, 705–719. doi: 10.1111/ejn.12453, 24329933 PMC4916881

[ref2] Chen Q. Cai Y. Zhu X. Wang J. Gao F. Yang M. . (2022). Edaravone dexborneol treatment attenuates neuronal apoptosis and improves neurological function by suppressing 4-HNE-associated oxidative stress after subarachnoid hemorrhage. Front. Pharmacol. 13:848529. doi: 10.3389/fphar.2022.848529, 35529450 PMC9068884

[ref3] Cheng Q. Yang W. Liu K. Zhao W. Wu L. Lei L. . (2019). Increased sample entropy in EEGs during the functional rehabilitation of an injured brain. Entropy 21:698. doi: 10.3390/e21070698, 33267412 PMC7515210

[ref4] Delcamp C. Srinivasan R. Cramer S. C. (2024). EEG provides insights into motor control and neuroplasticity during stroke recovery. Stroke 55, 2579–2583. doi: 10.1161/strokeaha.124.048458, 39171399 PMC11421965

[ref5] Ferreira L. O. Mattos B. G. de Jóia Mello V. Martins-Filho A. J. da Costa E. T. Yamada E. S. . (2021). Increased relative delta bandpower and delta indices revealed by continuous qEEG monitoring in a rat model of ischemia-reperfusion. Front. Neurol. 12:645138. doi: 10.3389/fneur.2021.645138, 33897602 PMC8058376

[ref6] GBD 2019 Stroke Collaborators (2021). Global, regional, and national burden of stroke and its risk factors, 1990–2019: a systematic analysis for the Global Burden of Disease Study 2019. Lancet Neurol. 20, 795–820. doi: 10.1016/s1474-4422(21)00252-0, 34487721 PMC8443449

[ref7] Hu R. Liang J. Ding L. Zhang W. Liu X. Song B. . (2022). Edaravone dexborneol provides neuroprotective benefits by suppressing NLRP3 inflammasome-induced microglial pyroptosis in experimental ischemic stroke. Int. Immunopharmacol. 113:109315. doi: 10.1016/j.intimp.2022.109315, 36279668

[ref8] Huang Y. Zhang X. Zhang C. Xu W. Li W. Feng Z. . (2022). Edaravone dexborneol downregulates neutrophil extracellular trap expression and ameliorates blood-brain barrier permeability in acute ischemic stroke. Mediat. Inflamm. 2022:3855698. doi: 10.1155/2022/3855698 PMC9410976 36032782

[ref9] Khoshnam S. E. Winlow W. Farzaneh M. Farbood Y. Moghaddam H. F. (2017). Pathogenic mechanisms following ischemic stroke. Neurol. Sci. 38, 1167–1186. doi: 10.1007/s10072-017-2938-1, 28417216

[ref10] Liu S. Guo J. Meng J. Wang Z. Yao Y. Yang J. . (2016). Abnormal EEG complexity and functional connectivity of brain in patients with acute thalamic ischemic stroke. Comput. Math. Methods Med. 2016:2582478. doi: 10.1155/2016/2582478 27403202 PMC4923597

[ref11] Liu R. Zhang L. Lan X. Li L. Zhang T.-T. Sun J.-H. . (2011). Protection by borneol on cortical neurons against oxygen-glucose deprivation/reperfusion: involvement of anti-oxidation and anti-inflammation through nuclear transcription factor κappaB signaling pathway. Neuroscience 176, 408–419. doi: 10.1016/j.neuroscience.2010.11.029, 21168474

[ref12] Ren Y. Wei B. Song X. An N. Zhou Y. Jin X. . (2015). Edaravone’s free radical scavenging mechanisms of neuroprotection against cerebral ischemia: review of the literature. Int. J. Neurosci. 125, 555–565. doi: 10.3109/00207454.2014.959121, 25171224

[ref13] Sun R. Wong W.-W. Gao J. Wong G. F. Tong R. K.-Y. (2021). Abnormal EEG complexity and alpha oscillation of resting state in chronic stroke patients. 2021 43rd Annual International Conference of the IEEE Engineering in Medicine & Biology Society (EMBC). 6053–6057. 10.1109/EMBC46164.2021.9630549 34892497

[ref14] Sun R. Wong W.-W. Wang J. Tong R. K.-Y. (2017). Changes in electroencephalography complexity using a brain computer interface-motor observation training in chronic stroke patients: a fuzzy approximate entropy analysis. Front. Hum. Neurosci. 11:444. doi: 10.3389/fnhum.2017.00444, 28928649 PMC5591875

[ref15] Tuo Q. Z. Zhang S. T. Lei P. (2022). Mechanisms of neuronal cell death in ischemic stroke and their therapeutic implications. Med. Res. Rev. 42, 259–305. doi: 10.1002/med.21817 33957000

[ref16] Wang Y. Qiu X. Y. Liu J. Y. Tan B. Wang F. Sun M. J. . (2023). (+)-borneol enantiomer ameliorates epileptic seizure via decreasing the excitability of glutamatergic transmission. Acta Pharmacol. Sin. 44, 1600–1611. doi: 10.1038/s41401-023-01075-w, 36973542 PMC10374614

[ref17] Watanabe T. Tanaka M. Watanabe K. Takamatsu Y. Tobe A. (2004). Research and development of the free radical scavenger edaravone as a neuroprotectant. J. Pharm. Soc. Jpn. 124, 99–111. doi: 10.1248/yakushi.124.99 15049127

[ref18] Wu J. Srinivasan R. Burke Quinlan E. Solodkin A. Small S. L. Cramer S. C. (2016). Utility of EEG measures of brain function in patients with acute stroke. J. Neurophysiol. 115, 2399–2405. doi: 10.1152/jn.00978.2015, 26936984 PMC4922461

[ref19] Xu J. Wang A. Meng X. Yalkun G. Xu A. Gao Z. . (2021). Edaravone dexborneol versus edaravone alone for the treatment of acute ischemic stroke: a phase III, randomized, double-blind, comparative trial. Stroke 52, 772–780. doi: 10.1161/STROKEAHA.120.031197, 33588596

[ref20] Yang X. Wang B. Wang Y. (2022). Keap1-Nrf2/ARE pathway-based investigation into the mechanism of edaravone dexborneol in cerebral infarction model neuroprotection. Cell. Mol. Biol. 68, 102–108. doi: 10.14715/cmb/2022.68.9.16, 36905267

[ref21] Zhang H. Zhu C. Zhou X. Wang L. Deng L. He B. . (2025). Edaravone dexborneol protected neurological function by targeting NRF2/ARE and NF-κB/AIM2 pathways in cerebral ischemia/reperfusion injury. Front. Pharmacol. 16:1581320. doi: 10.3389/fphar.2025.1581320, 40351418 PMC12062797

